# Hypofractionated stereotactic radiotherapy of limited brain metastases: a single-centre individualized treatment approach

**DOI:** 10.1186/1471-2407-12-497

**Published:** 2012-10-25

**Authors:** Bettina Märtens, Stefan Janssen, Martin Werner, Jörg Frühauf, Hans Christiansen, Michael Bremer, Diana Steinmann

**Affiliations:** 1 Radiation Oncology, Medical School Hannover, Carl-Neuberg-Str. 1, Hannover, 30625, Germany

**Keywords:** Brain tumours, hfSRT, SRS, Fractionation

## Abstract

**Background:**

We retrospectively report treatment results of our single-centre experience with hypofractionated stereotactic radiotherapy (hfSRT) of limited brain metastases in primary and recurrence disease situations. Our aim was to find the most effective and safe dose concept.

**Methods:**

From 04/2006 to 12/2010, 75 patients, with 108 intracranial metastases, were treated with hfSRT. 52 newly diagnosed metastases (48%), without up-front whole brain radiotherapy (WBRT), received hfSRT as a primary treatment. 56 metastases (52%) received a prior WBRT and were treated in this study in a recurrence situation. Main fractionation concepts used for primary hfSRT were 6-7x5 Gy (61.5%) and 5x6 Gy (19.2%), for recurrent hfSRT 7-10x4 Gy (33.9%) and 5-6x5 Gy (33.9%).

**Results:**

Median overall survival (OS) of all patients summed up to 9.1 months, actuarial 6-and 12-month-OS was 59% and 35%, respectively. Median local brain control (LC) was 11.9 months, median distant brain control (DC) 3.9 months and intracranial control (IC) 3.4 months, respectively. Variables with significant influence on OS were Gross Tumour Volume (GTV) (p = 0.019), the biological eqivalent dose (calculated on a 2 Gy single dose, EQD2, α/β = 10) < and ≥ median of 39 Gy (p = 0.012), extracerebral activity of the primary tumour (p < 0.001) and the steroid uptake during hfSRT (p = 0.03). LC was significantly influenced by the EQD2, ≤ and > 35 Gy (p = 0.004) in both uni- and multivariate Cox regression analysis. Median LC was 14.9 months for EQD2 >35 Gy and 3.4 months for doses ≤35 Gy, respectively. Early treatment related side effects were usually mild. Nevertheless, patients with a EQD2 >35 Gy had higher rates of toxicity (31%) than ≤35 Gy (8.3%, p=0.026).

**Conclusion:**

Comparing different dose concepts in hfSRT, a cumulative EQD2 of ≥35 Gy seems to be the most effective concept in patients with primary or recurrent limited brain metastases. Despite higher rates of only mild toxicity, this concept represents a safe treatment option.

## Background

Cancer patients develop brain metastases in 10-40% [1,2]. The aim of treatment is to provide disease control with a good quality of life, even though prolonged survival may not always be achieved [3]. Whole brain radiotherapy (WBRT) is considered to be the standard treatment option for multiple brain filiae [4,5]. Concerns about WBRT achieving only a limited treatment response and causing side-effects like cognitive and neurological deficits, and reduced quality of life [6-10] lead us to focus on options like stereotactic radiosurgery (SRS) or hypofractionated stereotactic radiotherapy (hfSRT) in cases of limited brain metastases. SRS is limited by the proximity to critical brain regions, the lesions’ dimension and is typically restricted to tumours ≤3 cm diameter (15 ccm) [11]. Large metastases and irregular contrast enhancement have been shown to correlate with an inferior outcome after SRS [12,13] or enhanced side effects [14,15]. Different study groups used hfSRT in order to overcome limitations of SRS, yielding similar tumour control and providing radiobiological advantages [16-20]. In 2006 we initiated the application of hfSRT whenever highly focussed stereotactic intracranial radiotherapy was indicated but tumour size or localization rendered a single-time treatment impossible.

HfSRT of more than one metastasis was carried out either simultaneously or successively in a primary setting or in a recurrent situation. The aim of this retrospective analysis was to evaluate an efficient and safe dose concept of hfSRT for limited (1–4) brain metastases.

## Methods

### Patient characteristics

Between April 2006 and December 2010, 75 patients with 108 brain metastases were treated with hfSRT and retrospectively analysed. After interdisciplinary discussion a surgical treatment approach had been excluded due to comorbidity, age, or localization of the tumour. Diagnosis of brain metastases was based on pre-treatment magnetic resonance imaging (MRI). Only patients with Karnofsky performance status ≥70 were included. 41 patients (55%) received a primary definitive hfSRT of 52 newly diagnosed metastases (48%). 34 patients (45%) with 56 metastases (52%) were treated with hfSRT in a recurrence situation. Patient and treatment characteristics are summarized in Table 1 and 2.

**Table 1:** Patients characteristics

		**Total patients n=75**			
		**Primary n=41**		**Recurrence n=34**	
Age	Median (years), range	57.3	40.2-79.2	57.7	38.4-79.9
		n	[%]	n	[%]
Gender	male	20	48.8	15	44.1
	female	21	51.2	19	55.9
Primary tumour	Non-small cell lung cancer	22	53.6	11	32.4
	Breast cancer	5	12.2	12	35.3
	Malignant melanoma	6	14.6	3	8.8
	Renal cell carcinoma	4	9.8	2	5.9
	Small cell lung cancer	0	0	5	14.7
	Others	4	9.8	1	2.9
RPA-classification	1	7	17.1	13	38.2
	2	34	82.9	21	61.8
	3	0	0	0	0
Extracranial tumour status	in remission	5	12.2	11	32.4
	detectable, stable disease	10	24.4	8	23.5
	progressive	26	63.4	15	44.1
Metastases treated with hfSRT (patients)	1	32	78.0	19	55.9
	2	7	17.1	9	26.5
	3	2	4.9	5	14.7
	4	0	0	1	2.9
Time between diagnosis of primary tumour and brain metastasis	Median (months), range	12.5	0-166.4	12.3	0-337.7
Time between diagnosis of brain metastasis and start of hfSRT	Median (month), range	1.4	0.2-39.9	11.4	0.5-37.9
Intervall between WBRT and start of hfSRT	Median (month), range	0	0	12.7	0.5-28.8

**Table 2:** Treatment characteristics

	**Total metastases (108)**		**Primary**	**Range**	**Recurrence**	**Range**
			**m=52**		**m=56**	
GTV	Median (ccm)		1.0	0.1-19.0	2.0	0.1-29.2
PTV	Median (ccm)		4.7	1.1-41.0	9.2	1.6-62.4
Brain volume treated with 4 Gy (V_4Gy)_	Median (ccm)		18.4	0-55.3	16.6	0-68.7
Duration of hfSRT	Median (days)		15	8-32	16	5-23
Recurrence situation	WBRT dose concepts					[%]
	- 10x3 Gy		0		27	48.2
	- 15x2.5 Gy		0		11	19.7
	- 15x2 Gy		0		4	7.1
	- 20x2 Gy		0		8	14.3
	- others		0		6	10.7
			m	[%]	m	[%]
Localization of brain metastases	frontal/frontoparietal		15	28.8	13	23.2
	temporal		1	1.9	6	10.7
	parietal/occipital		13	25.0	15	26.8
	central brain		7	13.5	7	12.5
	brainstem		3	5.8	2	3.6
	cerebellum		8	15.4	11	19.6
	others		5	9.6	2	3.6
Dose concepts	EQD2 (Gy)	median GTV (ccm)				
10x3 Gy	33	9.29	1	1.9	3	5.4
5x4 Gy	23	4.00		0.0	3	5.4
7x4 Gy	33	4.70		0.0	11	19.6
8x4 Gy	37	2.75	2	3.8	4	7.1
9x4 Gy	42	5.56		0.0	2	3.6
10x4 Gy	47	4.50	7	13.5	2	3.6
4x5 Gy	25	4.00		0.0	1	1.8
5x5 Gy	31	2.00		0.0	5	8.9
6x5 Gy	38	1.00	10	19.2	14	25.0
7x5 Gy	44	0.87	22	42.3	4	7.1
5x6 Gy	40	0.76	10	19.2	7	12.5
total		1.46	52	100.0	56	100.0

The need of ethical approval is waived because of the retrospective character of the study. Authorization for the use of patient data was given to the authors by the Head of the Departement of Radiooncology of the Medical School in Hannover.

### Radiotherapy

All patients were informed about radiotherapeutic alternatives, in detail. If hfSRT was chosen, repeated MRI scans were conducted after completing radiotherapy, in order to detect early intracranial progress or radiotoxicity. All patients were immobilized using a tight thermoplastic stereotactic head mask. Helical-CT images of 2 mm slice thickness were fused with axial T1 weighted contrast enhanced MR images. Gross Tumour Volume (GTV) was defined as the contrast enhancing tumour, a 4 mm margin in all directions was added for definition of Planning Target Volume (PTV). Oncentra Masterplan Planning System (Nucleotron, Germany, 84 lesions) or the Brainlab iPlan System (Feldkirchen, 24 lesions) was used.

The chosen fractionation scheme depended on the size, number and site of the brain metastases as well as on the ‘re-irradiation’ factor in a recurrent situation. Single doses of 5 to 6 Gy were aimed in primary setting, single lesions, small GTV (< 2 ccm) and in uncritical regions. If normal brain volume receiving more than 4 Gy (V_4Gy_) exceeded 23 ccm, the single dose was reduced to 4 Gy to prevent toxicity [17]. For recurrence, generally a more restrictive fractionation concept (Equivalent dose in 2 Gy fractions (EQD2 < 40 Gy) was used. Dose concepts and associated mean GTV are summarized in Table 2.

The EQD2 makes different radiation schedules comparable and is calculated with the equation EQD2 = D × ([d + *α*/*β*/[2 Gy + *α*/*β*), considering the linear-quadratic model. D is the total dose, d is the dose per fraction and the α/β ratio is an experimentally defined value of tissues. Assuming an α/β ratio of 10 Gy for tumour cell kill, the EQD2 of the radiation concepts are 40 Gy (30 Gy in 5 fractions), 44 Gy (35 Gy in 7 fractions) and 47 Gy (40 Gy in 10 fractions), exemplarily (Table 2) [21].

Patient positioning was checked with an on-board imaging (IGRT) before irradiation by using a cone-beam CT (XVI, Elekta) and X-ray images (Iview, Elekta) for verification of the isocenter. Radiotherapy was carried out using a Linac with 6 MVX photons in four to six beams, and a multi leaf collimator with a leaf width of 1cm. For 24 lesions Brain Lab System with individual blends was used.

Between each fraction at least one day treatment interruption was provided.

### Follow-up

Patients were monitored on a regular basis by the treating radiation oncologist. Performance status, neurological symptoms, and steroid uptake were monitored. After a planned period of 6 to 12 weeks the first MRI control was performed. Further follow-up MRI scans were carried out in intervals of 2–3 months. Treatment failure was considered as occurrence of new or increased contrast enhancement in the irradiated area (with or without increased volumes). Early side effects, i.e. alopecia, fatigue or headache, were scored with the CTC-AE 3.0 scoring system. Late effects (> 90 days after hfSRT) were not considered as these could not be analysed systematically due to retrospective data collection.

### Statistical analysis

Analysed endpoints were local control (LC), distant brain control (DC), intracranial control (IC) and overall survival (OS). Local relapse was defined as occurrence of new or increasing contrast enhancement in follow-up MRI in the irradiation area, after involvement of an experienced neuroradiologist. Distant brain failure was defined as any new brain metastases beyond local relapse, intracranial failure as any intracranial progress including local relapse. Survival rates and univariate analysis were calculated by the Kaplan-Meier log-rank test. All events were measured from the first treatment day of hfSRT. The following variables were used: sex, age (<57.5y. vs. ≥57.5y.), primary (non-small cell lung cancer vs. other), RPA (recursive partitioning analysis) prognostic group (1 vs. 2), primary vs. recurrent treatment, GTV (<2.0 vs. ≥2.0 ccm), steroid uptake (yes or no), median V_4Gy_ (<16.7 ccm vs. ≥16.7 ccm), EQD2 (≤35 Gy vs. >35 Gy), median EQD2 (<39 Gy vs. ≥39 Gy), median time interval between diagnosis of the primary tumour and brain metastases (<12.5 vs. ≥12.5 months). Multivariate Cox Proportional Regression Analysis was performed only with variables significant in the univariate analysis.

T-test of independent samples was used to compare GTV of different dose concepts and to compare toxicity rates. All statistical analysis was performed using IBM SPSS Statistics 20. A p-value <0.05 was considered to be statistically significant.

## Results

### Overall survival and influencing factors

**Figure 1:**
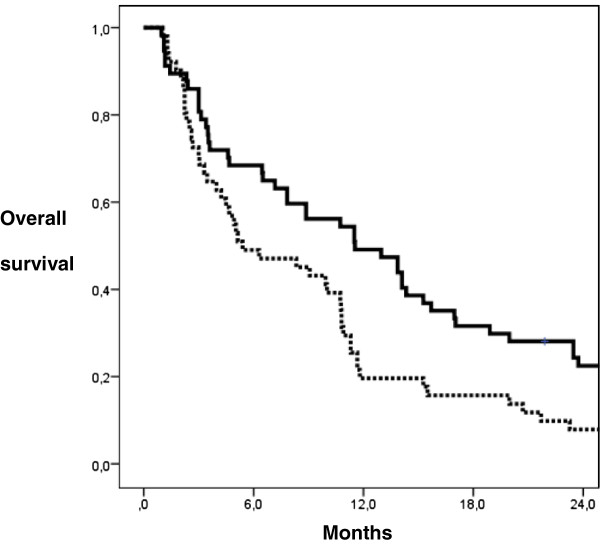
**Actuarial OS probability according to GTV calculated by Kaplan-Meier (log-rank test; p< 0.019).** GTV < 2 ccm (m=57): continuous line, GTV ≥ 2 ccm (m=51): dotted line.

Median OS summed up to 9.1 months with a median follow-up of 9.5 months (range 1.0-50.6 months). Median follow-up of patients being alive until August 2012 (n=5,7%) was 36.6 months (range 21.9-50.6 months). Actuarial 6- and 12-months-OS was 59% and 35%. Variables with significant influence on OS after multivariate analysis were the extracranial activity of primary tumour (p<0.001), the steroid uptake (p=0.03), the GTV (p=0.019) (Figure 1) and a total dose EQD2≥39 Gy (p=0.012). Detailed OS data is presented in Table 3.

**Table 3:** Actuarial overall survival according to potential factors and the result of univariate and multivariate analysis

	**No. metastases**	**1-year actuarial OS (%)**	**Median OS (months)**	**Univariate*p*value**	**Multivariate*p*value**
Gender				0.955	
male	55	35	9.9		
female	53	33	9.1		
Age				0.777	
< 57.5 years (median)	54	44	9.9		
≥ 57.5 years (median)	54	24	6.3		
NSCLC vs. Others				0.778	
NSCLC	43	40	7.2		
others	65	32	10.7		
RPA				**0.010**	
I	36	50	11.8		
II	72	28	4.7		
Extracerebral activity of primary tumor				**0.001**	**0.001**
not detectable	30	47	11.7		
stable	25	48	10.0		
progredient	53	23	4.0		
Objective of therapy				0.217	
primary	52	37	8.9		
recurrence	56	34	10.0		
Steroid uptake				**0.005**	**0.030**
yes	51	18	5.4		
no	57	51	13.0		
GTV				**0.004**	**0.019**
< 2 ccm	57	49	11.5		
≥ 2 ccm	51	20	5.4		
EQD2 (35 Gy)				**0.021**	0.298
≤ 35 Gy	24	13	3.6		
> 35 Gy	84	42	10.7		
EQD2 (median 39 Gy)				**0.009**	**0.012**
< 39 Gy	54	26	7.2		
≥ 39 Gy	54	44	11.5		
V4 Gy (median 16.7 ccm)				0.271	
< 16.7 ccm	56	32	7.2		
≥ 16.7 ccm	52	38	9.1		
Time between diagnosis of primary and diagnosis of brain metastases				0.092	
< 12.5 months	52	30	9.1		
≥ 12.5 months	56	40	8.9		

### Local and intracranial control

After a median of 2.2 months (range: 0.3-10.5 months) patients received the first follow-up MRI scan. Two patients (2.7%) were lost to follow-up and fourteen patients (18.7%) died within 90 days (range 13–75 days) of hfSRT and before the first follow-up imaging was carried out. 88 lesions (81.5%) were evaluated.

Imaging was done in 56 cases within 40–90 days after the end of hfSRT. 29 metastases (51.8%) showed a complete remission, 20 (35.7%) a partiell remission and 7 (12.5%) a progress.

**Figure 2:**
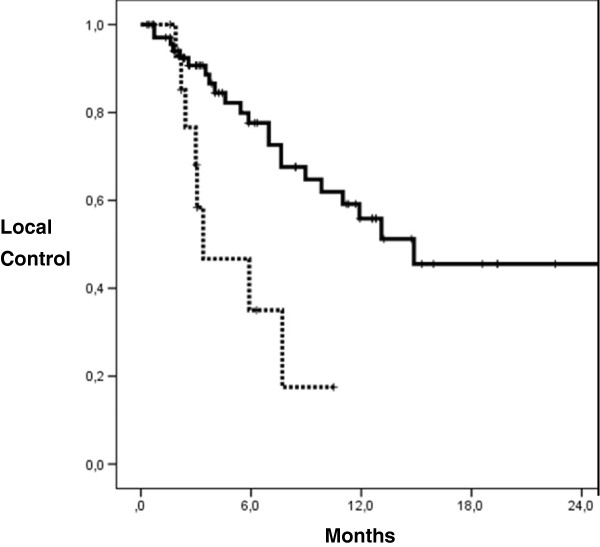
**Actuarial LC probability according to EQD2 calculated by Kaplan-Meier (log-rank test; p=0.004).** EQD2 > 35 Gy (m=73): continuous line, EQD2 ≤ 35 Gy (m=15): dotted line.

Local control in the irradiated area after 6 and 12 months was 73% and 52%, respectively, median LC was 11.9 months. The EQD2 significantly influenced LC (p=0.004, multivariate analysis). Median LC was 14.9 months for EQD2 >35 Gy and 3.4 months for doses ≤35 Gy. Actuarial LC rates for biological irradiation doses > and ≤35 Gy were 80%/44% after 6 months and 57%/22% after 12 months, respectively (Figure 2). The median EQD2 of < and ≥39 Gy was not a significant factor influencing LC.

LC analysis of different dose concepts showed a 6- and 12-months LC of 90%/66% for the 6x5 Gy concept. LC for 5x6 Gy was 83%/63%, 73%/42% for 7x5 Gy and 64%/21% for 10x4 Gy, respectively. Differences in LC were not significant.

Distant brain control (outside the irradiated area) after 6 and 12 months was 51% and 35%, median DC was 3.9 months. Fifteen patients with primary treated metastases suffered from a distant brain relapse, four had multiple metastases (≥3) and eleven developed limited (1–2) metastases. Two patients with distant brain relapse were treated with hfSRT, nine with WBRT, one got further chemotherapy and one had a surgical resection. Treatment of two patients was not known.

Intracranial control (includes local and distant brain control) was 41% and 20%, median being 3.4 months. No other variables with significant influence on DC, IC, LC and OS could be identified.

### Survival data of recurrence hfSRT

In recurrent situations 56 metastases were treated with a median EQD2 of 38 Gy while in comparison 52 metastases in primary situation were treated with a median EQD2 of 44 Gy.

The aspect of primary or recurrence hfSRT did not have a statistically significant influence on OS or on LC. Median OS of primary hfSRT was 8.8 months, while in recurrence situations it was 10 months (p=0.217 in univariate and p=0.124 in multivariate Cox regression analysis). 6- and 12-months-OS-rates for primary hfSRT were 62%/37% and for recurrence hfSRT they were 57%/34%. 6- and 12-months-rates of LC, DC and IC for primary hfSRT were 75%/55%, 56%/51% and 46%/30% while for recurrence hfSRT they amounted 71%/49%, 46%/25% and 38%/16%, respectively.

### Toxicity

For proper interpretation of toxicity results, we would like to point out, that 36 (48.0%) patients were treated with chemotherapy during the 3-month time period before the start of hfSRT. Furhtermore, it should be considered that 40 patients (53.3%) received steroids during radiotherapy. The median dose of dexamethasone at the end of radiotherapy was 4 mg per day.

Only mild early toxicity (CTC-AE v. 3.0 grade 1) was observed: alopecia (9 patients), fatigue (8 patients), headache (6 patients), nausea (2 patients) or mucositis (2 patients). One patient with hfSRT to four brain metastases, treated with a molecular therapy (Sunitinib) until the start of radiation, showed short term memory loss and amnesic aphasia. Another patient with chemotherapy just until the start of hfSRT suffered from vision disorder.

In only 2 cases out of 24 with a radiation dose EQD2 ≤35 Gy (8.3%) a toxicity was seen compared to 26 cases out of 84 with an EQD2 >35 Gy (31%, p=0.026).

The GTV (< and ≥2 ccm) and the a brain volume treated with 4 Gy (V_4Gy)_ (< and ≥ median of 16.7 ccm or < and ≥ 23 ccm) had no significant influence on toxicity (29.8% vs. 21.6%, p=0.328; 30.4% vs. 21.2%, p=0.275; 31.2% vs. 17.1%, p=0.149).

One case of radiation necrosis was discussed in the follow-up analysis. This patient with small cell lung carcinoma was initially treated with a prophylactic WBRT with 30 Gy (15x2 Gy). After 14 months the MRI showed 3 metastases of the brain, each of them was treated with hfSRT, the cerebellar metastasis was treated with a total dose of 40 Gy (10x4 Gy, EQD2 = 47 Gy). This resulted in a cumulative dose of 77 Gy in the cerebellum. The suspicion of necrosis was raised after a progress of the cerebellar metastasis with additional calcification showing 6 months later. Simultaneously, the patient suffered from cephalgia, emesis, vertigo and motor dysfunction. She died 3 months later after implantation of an Omaya reservoir and subsequent treatment with chemotherapy and steroids.

## Discussion

This study retrospectively analyses therapeutic results in 75 patients treated with hfSRT to limited brain metastases in a primary, or recurrence, situation. Since 2006 we are offering this option to patients who suffer from limited brain metastases and where surgery or SRS is not a suitable treatment option. We applied different dose concepts dependent on primary or recurrence situation, localization and volume of brain lesion. Dose concepts with a total EQD2 >35 Gy achieved best local control rates with acceptable toxicity. 12-months LC rates of 52% are lower in comparison to other hfSRT studies or SRS probably because we used very different dose concepts [16,22-27].

The aim of our study was to find the most suitable regime with respect to disease control and side effects. Fahrig et al. also compared different dose concepts like 5x6-7 Gy, 7x5 Gy and 10x4 Gy and achieved 12 months OS of 43%, 60% and 67%, respectively [28]. They preferred the 10x4 Gy fractionation, depending on the size and localization of the metastases, and detected no adverse side effects. However, a trend towards higher rates of complete remission in patients with brain metastases was seen treated with 5x6–7 Gy or 7x5 Gy in comparison with 10x4 Gy [28]. We preferred using restrictive dose concepts (EQD2 < 40 Gy) in patients with prior WBRT as well as in patients with high tumour volume or tumour proxicity to critical structures. The median EQD2 for GTV < 2 ccm was 40 Gy and for GTV ≥ 2 ccm 38 Gy. The GTV was significantly higher for metastases treated with a single dose of 3–4 Gy (median 4.76 ccm) than with 5–6 Gy (median 1.00 ccm, p < 0.001). Recent studies yielded LC rates of 58.6% after 12 months in mean volumes of 8 ccm (24 Gy in 3 fractions) [22] and 76% in median volumes of 4.23 ccm (5x6 Gy after prior WBRT, otherwise 5x7 Gy) [17]. Aoyama et al. found a significant lower tumour control rate for tumours >3 ccm (35 Gy in 4 fractions) [29]. Nevertheless, above mentioned hfSRT studies included volumes higher than 3 ccm and showed good results.

 Conclusions can be drawn from our study results only to a limited extent because of the relatively small number of patients included, it being a mono-institutional series and the potential risk of selection biases due to the retrospective study design. However, we consider the results to be valuable with regard to the objective of our analysis. Conclusions of our analysis are also limited due to high diversity of dose concepts (Table 2). In our study only 48% could be classified into dose concepts defined by Fahrig et al. [28]. Therefore, we could not see significant difference for a specific dose concept for LC or OS.

Furthermore, we detected a significant influence of the EQD2 (p = 0.004) on LC (14.9 months for doses >35 Gy and 3.4 months for doses ≤ 35 Gy) in this study.

An EQD2 >35 Gy is associated with better control rates along with higher but acceptable toxcitiy. Therefore, we consider it is most effective for tumour control.

In SRS higher absolute doses (24 Gy) are associated with higher LC (85%) after 12 months (compared to 45-49% with 15–18 Gy) [30]. For brain metastases ≤2 ccm SRS studies found that a single time 20 Gy application seems to render superior results [31,32].

Rades et al. achieved higher LC rates with upfront WBRT (77%) than with SRS alone (49%), and thereby showed a benefit of up-front WBRT [33].

Half of our patients (45%) were treated in a recurrence situation after prior WBRT. LC was not significantly different (49% after 12 months) in comparison to patients who received primary treatment (55% after 12 months). Lindvall et al. combined WBRT and hfSRT (30 Gy in 10 fractions and 17 Gy in 1–3 fractions) in 11 patients and compared them to 44 patients with hfSRT (40 Gy in 5 fractions) alone. They showed high LC rates of 100% and 84% in a short observation time (mean 3.7 months) [19].

Nevertheless, GTV significantly influenced OS in our study (< 2 ccm median 11.5 months ≥2 ccm median 5.4 months). Ernst-Stecken et al. defined GTV and PTV volume above 6 ccm and 13 ccm as negative prognostic factor for OS [17].

According to the results of Ernst-Stecken et al. brain volume receiving >4 Gy per fraction should not exceed 23 ccm [17]. Takening this into account, overall adverse effects were only mild in our study. None of our patients suffered from seizures classified as a higher grade side effect. In contrast, 11% of patients suffered of seizures within 3 months after SRS [23].

A recurrence situation after WBRT or hfSRT has not shown a statistically significant influence on LC, IC or OS in our and other studies [22]. Overall survival for hfSRT in primary and recurrence situations was median 8.8 months and 10 months, respectively. The actuarial OS of 35% one year after treatment with hfSRT is comparable to the reported SRS series (30-50%) [16,23,25-27] and hfSRT studies (25%) [22]. Different studies compared SRS alone with WBRT plus a SRS boost. The omission of WBRT in the initial management of patients who underwent SRS alone did not compromise survival or intracranial control [24,27,34,35]. De Potter et al. [36] achieved a DC at 1 year of 75%. They used 5x6 Gy as a boost in addition to WBRT. Patients with primary hfSRT treatment in our study achieved a DC of only 51% after one year and 60% of them had a WBRT as salvage therapy. Similar DC results of 36% without up-front WBRT were presented by Narayana et al. [37].

Therefore, up-front WBRT is worth discussing and not simply expendable to avoid neurotoxicity of the normal brain tissue. Regular MR imaging is necessary to detect cerebral progress as soon as possible. Patients with limited brain metastases should be clearly informed about all possible advantages and disadvantages of the different therapy options.

## Conclusion

HfSRT to limited brain metastases is a non invasive therapy in primary and recurrence situations, and provides a reasonable tumour control and survival benefit. A total EQD2 >35 Gy is associated with better tumour control rates and with higher but acceptable toxcitiy. Therefore, it is most effective for control of limited brain metastases.

## Competing interests

The authors declare that they have no competing interests.

## Authors’ contributions

MB and DS participated in the design of the study. BM and DS performed the statistical analyses. All authors provided study material and were involved in manuscript writing; they read and approved the final manuscript. BM and DS drafted the manuscript.

## Pre-publication history

The pre-publication history for this paper can be accessed here:

http://www.biomedcentral.com/1471-2407/12/497/prepub
